# Nanographene and Graphene Nanoribbon Synthesis via Alkyne Benzannulations

**DOI:** 10.3390/molecules24010118

**Published:** 2018-12-30

**Authors:** Amber D. Senese, Wesley A. Chalifoux

**Affiliations:** Department of Chemistry, University of Nevada, Reno, 1664 N. Virginia St., Reno, NV 89557, USA; amber.senese@gmail.com

**Keywords:** alkyne, benzannulation, nanographene, polycyclic aromatic hydrocarbon, graphene nanoribbon

## Abstract

The extension of π-conjugation of polycyclic aromatic hydrocarbons (PAHs) via alkyne benzannulation reactions has become an increasingly utilized tool over the past few years. This short review will highlight recent work of alkyne benzannulations in the context of large nanographene as well as graphene nanoribbon synthesis along with a brief discussion of the interesting physical properties these molecules display.

## 1. Introduction

The term “nanographenes (NGs)” has recently become a popular term used to describe relatively large polycyclic aromatic hydrocarbons (PAHs) [1]. These molecules represent discrete sections of graphene, a material with its own interesting chemical and physical properties [2,3]. There has been increasing interest in the synthesis of NGs due to their use in applications such as organic light-emitting diodes (OLEDs), organic field-effect transistors (OFETs), and in solar-cell applications [4,5,6,7,8,9,10,11,12]. The attraction to NGs is that these “soft” materials have useful physical properties that can be easily tuned through structurally changes and/or substitution. A related class of compounds made of long, narrow strips or ribbons of graphene, known as “graphene nanoribbons (GNRs),” with large aspect ratios, have shown ideal semiconducting properties for potential use in OFETs [13,14,15]. The development of bottom-up synthetic tools for the production of GNRs has been an area of recent interest for the purpose of imparting solubility and tuning of the material properties. This short review will discuss the state of the art for NG and GNR synthesis with a focus on alkyne benzannulations [16] as the key chemical transformation.

## 2. Catalyst- and Reagent-Free Alkyne Benzannulations

### 2.1. Alkyne Benzannulations via Pyrolysis

**Scheme 1:**
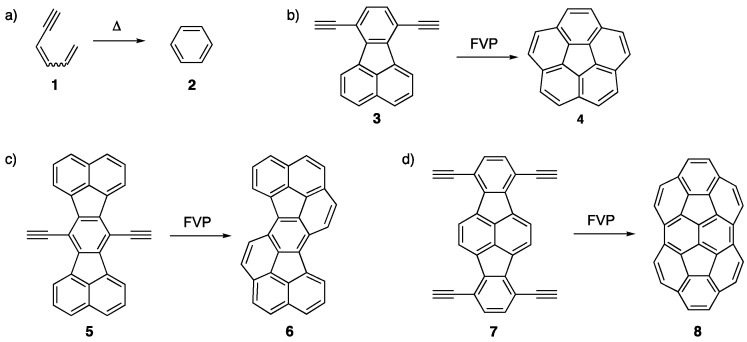
(**a**) Benzene **2** from the pyrolysis of 1,3-hexadien-5-yne **1** [17]. (**b**) FVP of **3** to produce corannulene **4** [20]. Two-fold [21] and four-fold alkyne benzannulations [22] to afford bowl-shaped NGs (**c**) **6** and (**d**) **8**, respectively.

In 1969, Hopf and Musso showed that pyrolysis of *cis*-1,3-hexadien-5-yne **1** leads to the formation of benzene **2** (Scheme 1a) [17]. Pyrolysis of analogous molecules containing the 1,3-hexadien-5-yne substructure have led to small NGs such as naphthalenes [18,19]. Scott and coworkers utilized flash vacuum pyrolysis (FVP) for the conversion of **3** to corannulene **4**, a bowl-shaped NG, which involves a two-fold alkyne benzannulation reaction (Scheme 1b) [20]. Shortly after this, others adopted this procedure to produce other non-planar NGs such as **6** and **8** (Scheme 1c,d) [21,22]. 

### 2.2. Alkyne Benzannulatinos via Photocyclizations 

**Scheme 2:**
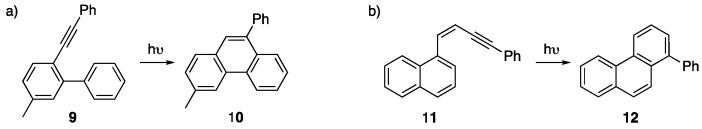
Alkyne benzannulation of (**a**) 2-ethynylbiphenyl derivatives **9** [23] and (**b**) 1,4-diaryl-1-buten-3-ynes **11** [24,25] via a photocyclization reaction to afford phenanthrene products **10** and **12**.

Photochemical alkyne benzannulations to afford large NGs are rare but there have been a few examples reported of smaller NGs being formed this way. For example, photocyclization of 2-ethynylbiphenyls such a **9** (Scheme 2a) [23] and 1,4-diaryl-1-buten-3-ynes **11** (Scheme 2b) [24,25] is known to afford phenanthrene products **10** and **12**. With the success of photochemical alkyne benzannulations of smaller NG systems, the relatively underexplored area of larger NGs synthesized in this manner appears to be ripe with low-lying fruit.

## 3. Alkyne Benzannulations Promoted by Electrophilic Reagents

### Iodonium Salt- or Iodine Monochloride (ICl)-Induced Alkyne Benzannulations 

**Scheme 3:**
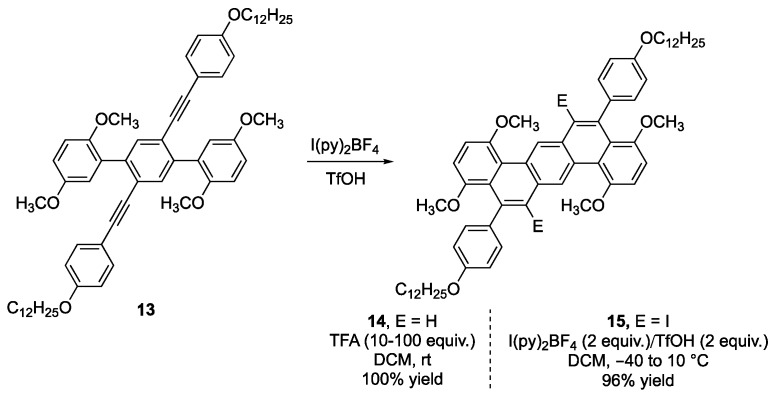
Electrophilic benzannulation with either an iodonium salt or a Brønsted acid [28].

Electrophilic iodine reagents are an excellent way of cyclizing alkynes with neighboring aryl groups to produce a new benzene ring [26]. This methodology has the added advantage of providing a functional handle in the product for further chemical elaboration. This chemistry was first developed in 1988 by Barluenga and coworkers in which they used I(py)_2_BF_4_ as an electrophilic iodine source in the presence of TfOH to produce iodocyclohexene products from 1,4-diphenyl-1-butyne [27]. Swager and coworkers utilized this reagent system to cyclize terphenyl derivative **13** to give a mixture of halogenated and non-halogenated NG products **14** and **15** (Scheme 3) [28]. If the incorporation of an iodide group in the final product is desired, using equal equivalents of TfOH and I(py)_2_BF_4_ resulted in good yields of the halogenated product **15**. 

**Scheme 4:**
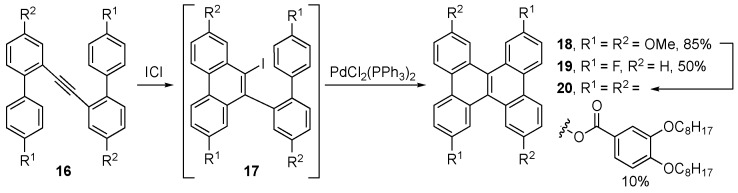
ICl-induced benzannulation used in the synthesis towards dibenzo[g,p]chrysene derivatives [29].

Liu and coworkers took advantage of an ICl-induced alkyne benzannulation of bis(biaryl)acetylenes **16** to arrive at iodide-functionalized phenanthrene intermediates **17** (Scheme 4) [29]. These intermediates are poised for a palladium-catalyzed intramolecular direct arylation to arrive at dibenzo[*g,p*]chrysenes in good to excellent overall yield. This route allowed for the synthesis of this class of NGs bearing both electron-rich (**18**) and electron-poor (**19**) substituents. This was important as Liu and coworkers were able to demonstrate that dibenzo[*g,p*]chrysenes functionalized with electron-withdrawing groups have larger HOMO/LUMO energy gaps (3.10–3.18 eV) and lower quantum yields (Φ = 12.4–16.8%) than derivatives with electron-donating groups (2.84–2.91 eV, Φ = 26.0–48.7%) [30]. They were also able to convert **18** to a liquid crystalline derivative **20** in 10% overall yield and this material displayed reasonable hole and electron transport in thin films. This work nicely demonstrates that physical properties of dibenzo[*g,p*]chrysene can be tuned through substituent effects.

**Scheme 5:**
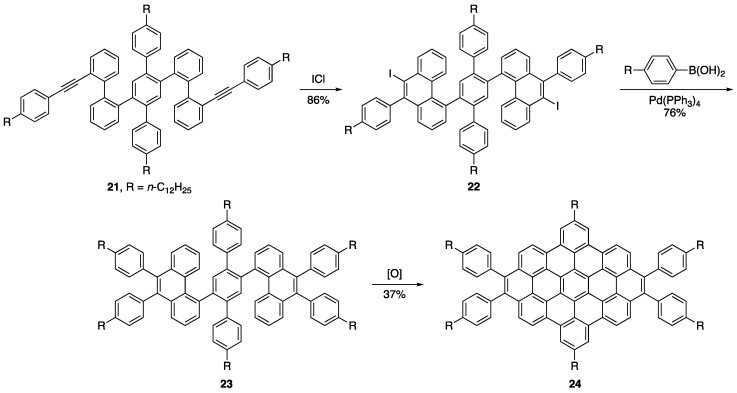
Müllen and coworkers synthesis of zigzag NGs [31].

The Müllen group recently used an ICl-induced two-fold alkyne benzannulation of compound **21** to generate two new iodinated phenanthrene units in product **22** (Scheme 5) [31]. They demonstrated the crosscoupling utility of these intermediates by generating compound **23**, which was further oxidized under Scholl conditions [32] to afford zigzag nanographenes **24**. The formation of NG intermediates with useful coupling handles has also been used towards the formation of conjugated polymers [33].

**Scheme 6:**
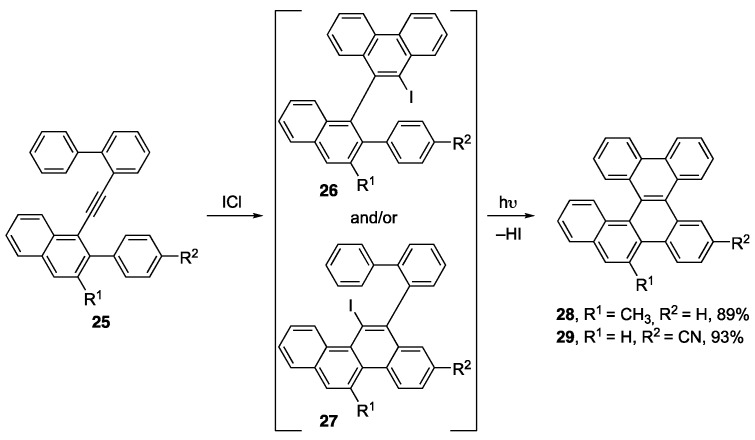
Synthetic route to [5]helicene-like compounds [35].

Molecular structure can greatly affect the properties exhibited by the bulk material, hence, it is important to understand how subtle changes to the molecular structure affect the HOMO/LUMO energy gaps, crystal packing, or UV-vis absorption and emission [34]. The way in which the twisted molecular structure of helicenes pack was of interest to the Alabugin group, who made [5]helicene-like compounds under metal-free conditions [35]. The reaction proceeds via an ICl-promoted alkyne benzannulation of **25** to afford either iodophenanthrene **26**, iodochrysene **27**, or a mixture of both (Scheme 6). This mixture was subjected to a cyclodehydroiodination reaction to afford [5]helicene derivatives **28** and **29**, which were the two derivatives that afforded single crystals suitable for X-ray crystallography. It was found that the 3D crystal architecture significantly varied from the placement of the functional groups around the [5]helicene skeleton, but the calculated HOMO/LUMO energy gaps remained quite similar in value for most of the [5]helicenes synthesized in the study (2.51–2.68 eV).

Electrophilic iodine-mediated alkyne benzannulations have proven to be an excellent tool for the synthesis of functionalized nanographenes [29,31,36,37,38]. The products are often achieved in high yields and provide a useful handle for further chemical transformations.

## 4. Radical-Mediated Alkyne Benzannulations

**Scheme 7:**
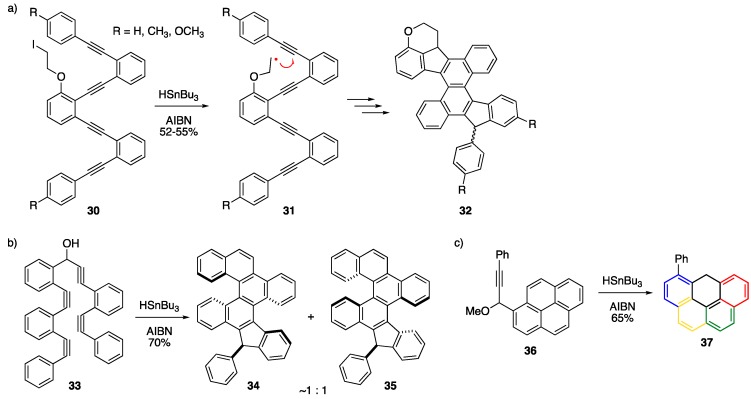
Radical mediated cascade alkyne benzannulations to arrive at (**a**) NG **32** [40], (**b**) helical NGs **34**/**35** [42], and (**c**) olympicene **37** [44].

The Alabugin group has done significant work over the past few years using high-energy alkyne-containing substrates to produce a host of NGs through thermodynamically “downhill” alkyne benzannulations [35,39,40,41,42,43,44]. Their recent work stems from their initial discovery that tin-mediated radical cascade cyclizations of alkynes can be used to afford substituted benzo[a]indeno[2,1-c]fluorenes and other products [45]. Alabugin and Byers later used this strategy to cleverly stitch together larger NGs **32** (Scheme 7a) [40]. In this single reaction, the authors impressively generate five new rings and five new carbon-carbon bonds in excellent yield (ca. 93% yield per step), which demonstrates the exceptional chemoselectivity of radical formation and regioselectivity of the initial cyclization on the internal alkyne, as depicted in Scheme 7a. The Alabugin group later came up with a “traceless” version of this reaction to get a range of NG products including the conversion of **33** to arrive at a mixture of helical systems such as **34** and **35** (Scheme 7b) [42]. The chemistry leaves the product with a useful SnBu_3_ handle that their group demonstrated could be further reacted with electrophiles or used in Stille crosscoupling reactions. In 2018, and perhaps fortuitously timed with the 2018 Olympic Winter Games, the Alabugin group extended their methodology to *peri*-cyclizations, allowing them to synthesize a number of NGs including olympicene derivative **37** (Scheme 7c) [44].

## 5. Acid-Mediated Alkyne Benzannulations

As demonstrated in Section 3, electrophilic reagents can be an effective way to promote 6-*endo*-*dig* cyclizations of alkynes to afford a new benzene ring and provide larger NG structures. Other electrophiles, such as acids, have also been effective in alkyne benzannulations over the past 30 years.

### 5.1. Brønsted Acid Catalyzed Alkyne Benzannulations

In Section 3 we highlighted how iodonium reagents can be used to promote alkyne benzannulations. In many cases, the *proto* (R = H, Scheme 3) product is desired and thus would require a second step of lithium-halogen exchange and protonation to arrive at the desired product [46]. Perhaps the simplest electrophile for effecting an alkyne benzannulations reaction to arrive directly at the *proto* product would be a proton. Some of the earliest work of alkyne benzannulations came from the Swager group and during an early study of electrophile-induced alkyne cyclizations using iodonium (Scheme 3) they noted that the use of TfOH or TFA alone very effectively promoted alkyne benzannulations to arrive at NG products [28]. This Brønsted acid-promoted benzannulation was hypothesized to occur through a *6-endo-dig* pathway due to the carbocation stability through resonance with an electron-rich arene moiety attached to the acetylene [46]. Having an electron-rich aryl group proved to be essential for the success of this type of alkyne benzannulation in other examples of NG syntheses [47,48,49,50,51,52]. 

**Scheme 8:**
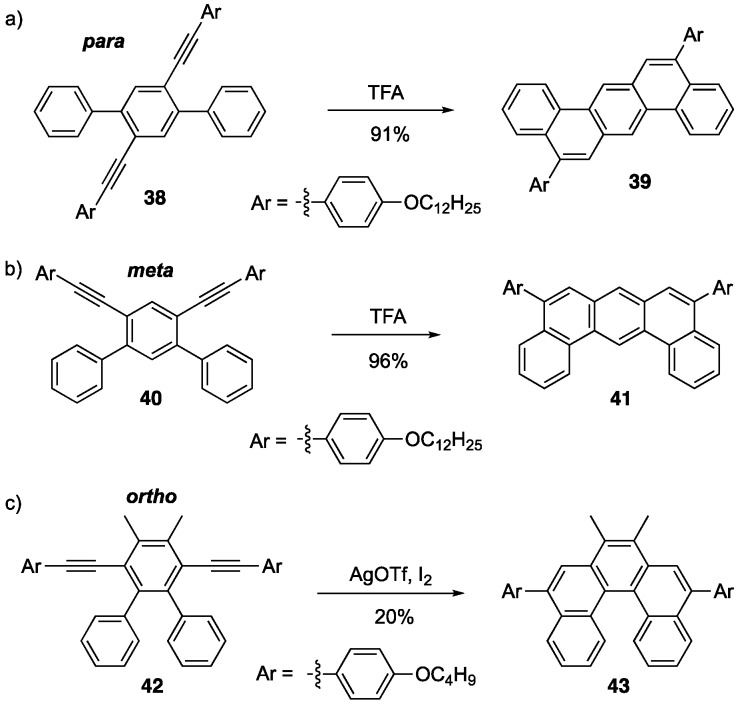
Brønsted acid-induced alkyne benzannulations of (**a**) *para*- (**38**), (**b**) *meta*- (**40**) and (**c**) *ortho*-substituted (**42**) terphenyl systems to afford NGs [46].

Swager and coworkers later explored their two-fold alkyne benzannulation reaction by looking at various substitution patterns of diethynylterphenyl systems in which the two aryl (Ar) groups participating in the alkyne benzannulation reaction were oriented *para-* (**38**), *meta-* (**40**) and *ortho-* (**42**) to each other to arrive at NGs **39**, **41**, and **43**, respectively (Scheme 8) [46]. Attempting to cyclize systems with more steric strain, as the case with the *ortho*-terphenyl system **42**, using Brønsted acids only provided trace amounts of desired products. The authors found that conducting the reaction in the presence of silver triflate and iodine resulted in the desired product in 20% yield. What was surprising is that there was no iodine detected in the NG product under these conditions. 

**Scheme 9:**
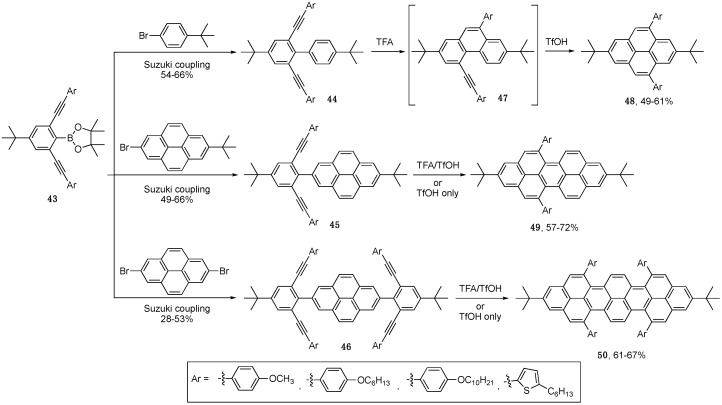
Brønsted acid-promoted two-fold and four-fold alkyne benzannulations to afford pyrenes **48**, peropyrenes **49**, and teropyrenes **50** [48,49].

The Chalifoux group has recently adapted Swager’s alkyne benzannulation conditions to afford a host of nanographenes [47,48,49,50]. In one study, they are able to generate diynes **44** and **45** or tetrayne **46** intermediates via Suzuki crosscoupling of boronic ester **43** and various brominated aromatics (Scheme 9) [48]. The idea here was to invoke two benzannulation reactions on both *ortho* positions of the same aryl moiety, whereas in Swager’s examples they invoke only one alkyne benzannulation on one *ortho* position of the neighboring aryl group [28,46]. The authors thought this would allow them to rapidly access larger fused NG systems with greater lateral conjugation. However, they found that under Swager conditions (TFA), cyclization of compound **44** only produced monocyclized phenanthrene product **47**, albeit quantitatively. The explanation for incomplete cyclization in this case, as compared to systems reported by Swager and coworkers [28,46], is that the first alkyne cyclization causes planarization to form rigid phenanthrene intermediate **47**. This leads to poor orbital interaction between the second (remaining) alkyne and the remaining *ortho* position, resulting in a much higher barrier for the second benzannulation. Adding excess amounts of TFA along with refluxing the reaction mixture did lead to pyrene product **48**, albeit in low yield. It was found that a stronger acid, such as TfOH, could be added to induce the second alkyne benzannulation to afford good yield of pyrene product **48**. This protocol proved to be an effective method for synthesizing highly soluble peropyrene and teropyrene products **49** and **50**, respectively. The two-step protocol of adding TFA followed by TfOH was useful for characterizing and studying the partially cyclized intermediates but the final nanographene products could be cleanly formed directly using just TfOH. The alkyne benzannulation reaction using Brønsted acid requires the presence of electron-rich ethynylaryl groups in the substrate and thus thiophene substituents are also tolerated [49]. The photophysical properties of these compounds were studied and, as expected, there is a red-shift in both the absorbance and emission in going from **48**, to **49**, to **50**. Peropyrene products **49** display high extinction coefficients of 5.6 × 10^4^ M^−1^·cm^−1^ at 467 nm and display green emission (λ_em_ = 486 nm) with quantum yields up to 64%. The teropyrenes **50** were significantly red-shifted with extinction coefficients of 2.8 × 10^5^ m^−1^cm^−1^ at 572 nm and have red emission (λ_em_ = 590 nm) with quantum yields up to 61%.

**Figure 1:**
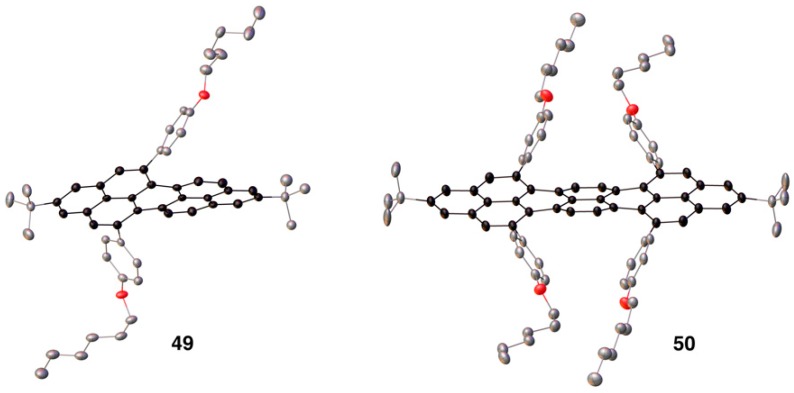
X-ray crystal structures of peropyrene **49** and teropyrene **50**.

Single crystals suitable of X-ray crystallographic analysis were obtained for compounds **49** and **50** (Ar = *p*-hexyloxyphenyl) (Figure 1). As can be seen, the NGs adopt a non-planar structure due to steric hindrance within the newly formed *bay* regions. This feature, along with the ability to functionalize the NGs using this methodology, is likely responsible for the high solubility of these compounds. What is interesting is that the twist of peropyrene **49** causes the molecule to be chiral in the solid-state whereas teropyrene **50** has a pseudo plane of symmetry. In solution, it was hypothesized that the inversion barrier was low for **49** making it impossible to isolate each enantiomer under ambient conditions. 

**Scheme 10:**
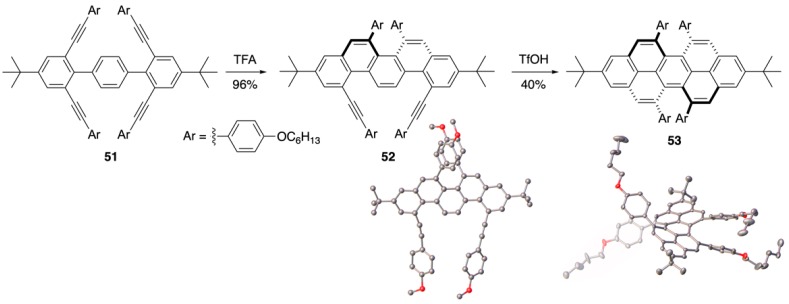
Four-fold alkyne benzannulation towards chiral peropyrenes **53** [50].

The Chalifoux group wondered if they could impart higher steric hinderance in the *bay* regions of a peropyrene through substitution in order to increase the inversion barrier and allow for isolation of the enantiomeric products. To probe the viability of synthesizing a persistently chiral peropyrene, the Chalifoux group synthesized tetrayne **51** and subjected it to their Brønsted acid-catalyzed alkyne benzannulation conditions (Scheme 10) [50]. Interestingly, using TFA alone resulted in a two-fold alkyne benzannulation in which the alkynes cyclized on the same side to produce chiral picene intermediate **52**, which was unambiguously confirmed by X-ray crystallographic analysis (Ar = *p*-MeOC_6_H_4_). This cyclization mode was supported by calculations that determined a lower barrier of about 2 kcal/mol for this product. Treating **52** with TfOH resulted in complete cyclization to afford the first ever reported chiral peropyrene **53**. The X-ray structure shows this molecule to be twisted from end-to-end by about 28°. The inversion barrier was determined to be ca. 29 kcal/mol making it possible to separate the enantiomers by chiral HPLC. An optical rotation [α]$\frac{25}{589}$ = value of + 1438 was determined for the pure enantiomer of (*P*,*P*)-**53**. Circular dichroism of **53** showed strong Cotton effects (Δε = ±100 M^−1^·cm^−1^ at 300 nm) and the compound also displayed circularly polarized luminescent properties, also observed in other helical NGs [53,54], making it potentially useful in chiroptic applications.

**Scheme 11:**
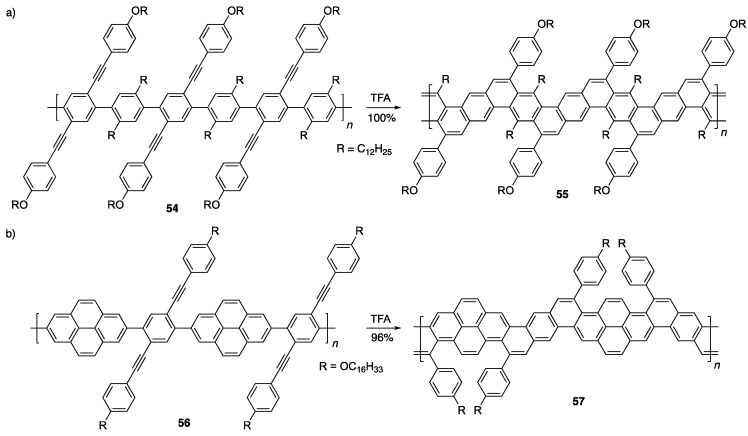
(**a**) Synthesis of GNRs **55** by Swager and coworker [28]. (**b**) Synthesis of expanded GNRs **57** by Wu, Zhao and coworkers [66].

Bottom-up synthesis of graphene nanoribbons (GNRs) has been attempted long before the discovery of graphene itself and research towards the synthesis of GNRs is still being conducted today [14,55,56,57,58,59,60,61,62,63,64]. In 1970, Stille et al. reported the first bottom-up synthesis of a GNR, however, these carbon-based ladder polymers suffered from considerable insolubility [65]. It wasn’t until 1994 that the Swager group realized the potential of using a Brønsted acid-promoted alkyne benzannulation of polymer **54** as a means of creating functionalized, soluble GNRs **55** (Scheme 11a) [28]. Their strategy involved the use of thoughtfully designed monomers that included both electron-donating groups to ensure clean cyclization and alkyl substitution (R) along the backbone to ensure good regiocontrol. The polymers had high molecular weights of Mn = 45,000–55,000 g/mol and displayed an absorption edge at 468 nm. About two decades later, Wu, Zhao and coworkers used a similar strategy to synthesize more conjugated and rigid pyrene-based GNRs **57** (Scheme 11b) [66]. As expected, they observed roughly a 100 nm red-shift in the absorption edge to ca. 550 nm relative to GNR **55**.

**Scheme 12:**
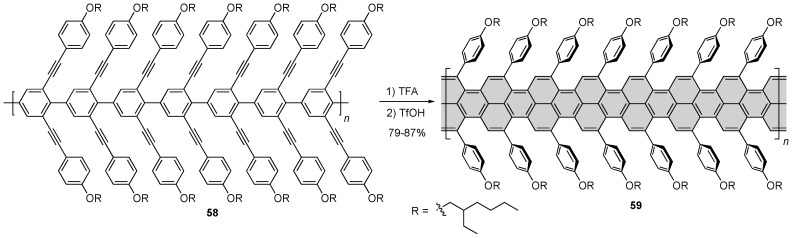
Soluble GNR **59** reported by Chalifoux and coworkers [67].

Taking a similar approach as with their nanographene synthesis (vide supra), Chalifoux and coworkers used a Brønsted acid-catalyzed alkyne benzannulation strategy to cyclize poly(2,6-dialkynyl-*p*-phenylene) **58** into a functionalized GNR **59** (Scheme 12) [67]. This flexible GNR was reported to have average lengths of 8 nm when the Suzuki polymerization was carried out in water and toluene as solvent and 35 nm when the Suzuki polymerization was carried out in water and THF as solvent. GNR **59** proved to be highly soluble in a number of common organic solvents allowing for its study by IR, Raman, NMR and UV/Vis/NIR spectroscopy. The GNR showed broad absorption in the visible and near-infrared spectrum with a large red-shift as compared to GNRs **55** and **57** (Scheme 11), displaying an absorption edge at 1200 nm, which corresponds to an optical bandgap of 1.03 eV.

The use of Brønsted acid-promoted alkyne benzannulations has proven to be a convenient route towards the synthesis of NGs and GNRs. However, the limitation that electron-rich ethynylaryl groups are needed for a successful 6-*endo*-*dig* cyclization under these conditions makes it a challenge to tune the electronic and optical properties through substituent effects.

### 5.2. π-Lewis Acid Catalyzed Alkyne Benzannulations

Over the last ca. 30 years, significant optimization of the alkyne benzannulation reaction has led to the discovery of a variety of reagents that promote cyclization in moderate to good yields under relatively mild reaction conditions. There have been numerous examples where π-Lewis acids have been used to activate an alkyne towards cyclization [68,69,70,71,72,73,74,75]. The η^2^-metal complex formed with the alkyne makes it susceptible to nucleophilic attack from the adjacent aromatic ring leading to a new C-C bond. In most cases, strongly deactivating groups, such as esters, attached to the alkyne were found to favor the *5-exo-dig* pathway, whereas activating groups almost exclusivity formed the *6-endo-dig* product [76]. With some understanding to mechanism of this reaction, researchers have begun taking advantage for the purpose of synthesizing large NG systems. 

#### 5.2.1. Transition Metal-Catalyzed Cyclizations

**Scheme 13:**
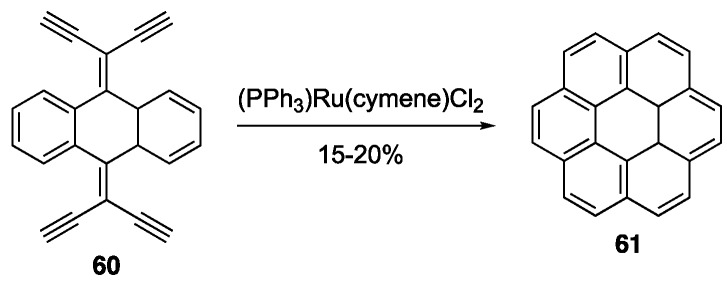
First synthesis of coronene **61** using a Ru(II)-catalyzed four-fold alkyne benzannulation [77].

Ruthenium catalysts have been shown to be effective at effecting alkyne benzannulations to afford NGs [68,75,76,77,78,79,80,81]. NGs doped with boron and silicon have also been produced using Ru(II) catalysts [82]. Scott and coworker demonstrated that two- and even four-fold alkyne benzannulations reactions using (Ph_3_P)Ru(cymene)Cl_2_ could produce a host of NGs, including coronene **61** (Scheme 13) [77]. Liu and coworkers were able to improve the yield of coronene and extend the scope of NGs synthesized in this way using a more reactive Ru(II) catalyst system [78]. 

**Scheme 14:**
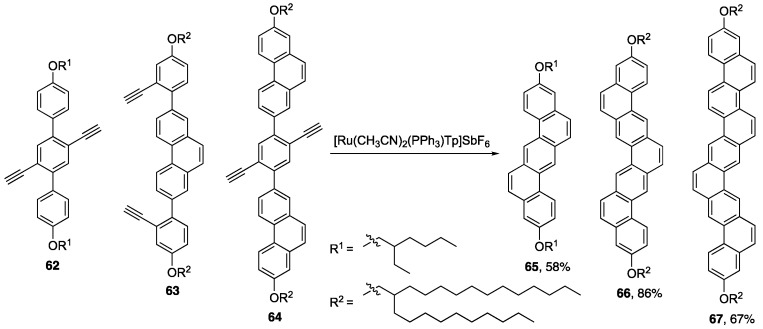
Synthesis of GNRs **65**–**67** via a Ru(II)-catalyzed two-fold alkyne benzannulation by Liu and coworkers [79]. [Tp = tris(1-pyrazolyl)borate)]

The Liu group was able to utilize Ru-catalysis to synthesize a series of NG ribbons of various lengths (Scheme 14) [79]. They later showed that substrates containing Ph_2_N groups were also tolerated in this reaction [80]. These second-generation ribbons were reported to have excellent quantum yields up to 96.3% with larger HOMO/LUMO energy gaps vs compounds **65**–**67**.

Though Ru(II) catalysis is useful for the formation of NGs and GNRs, it is limited to cyclizing compounds that contain terminal acetylenes. As one might imagine, planarization of the system leads to decreased solubility and thus incorporation of appropriate solubilizing groups elsewhere in the substrate needs to be done if using this methodology.

**Scheme 15:**
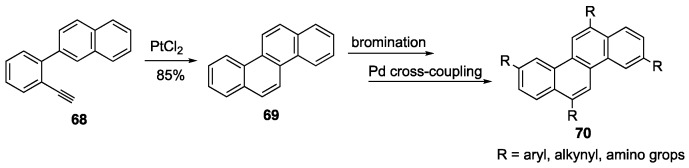
Pt-catalyzed alkyne benzannulation to afford chrysene **69** [83,87].

Alkyne benzannulations using Pt(II) catalysis has also become a popular method to arrive at various NGs [79,80,83,84,85]. For example, Liu and coworkers developed a four-step synthesis of chrysene **69** from cheap, commercially available starting materials with the key step involving a Pt(II)-catalyzed alkyne benzannulation of intermediate **68** (Scheme 15) [83]. Chrysene itself is a small NG that emits blue light at 383 nm with a quantum yield of 16% making derivatives of chrysene promising in OLED applications [86]. Studies on the effects that substituents have on the chrysene’s emission and quantum yield show that tetra- and disubstituted molecules still emit at blue wavelengths and the quantum yield can be increased to 49% [87]. Chrysene **69** can be further functionalized though bromination at the 3-, 6-, 9-, 12-postions to afford tetrabrominated chrysene which can undergo Pd-catalyzed crosscoupling to afford chrysene derivatives **70** [83,87]. An analogous version of this reaction has also been reported using microwave irradiation to arrive at other substitution patterns of chrysene [88].

**Scheme 16:**
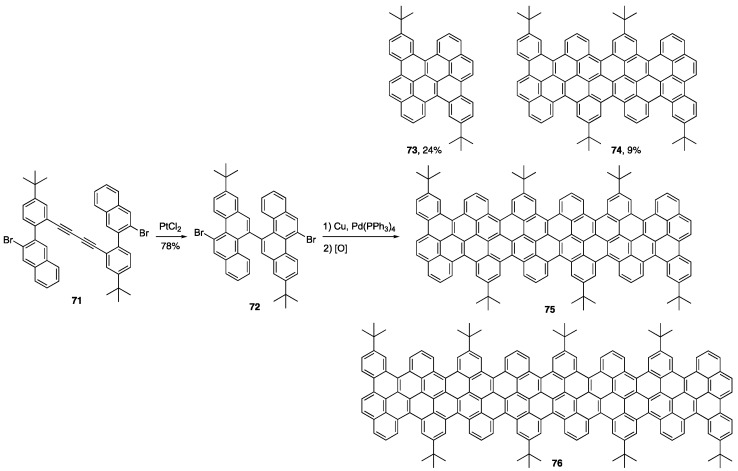
Cove-edge NGs produced from Pt(II)-catalyzed alkyne benzannulations [85].

In an attempt to create cove-edged GNRs using solution-phase chemistry, Müllen and coworkers employed a Pt(II)-catalyzed alkyne benzannulation of **71** to synthesize chrysene-based dimer **72** (Scheme 16) [85]. Dimer **72** was subjected to Ullmann coupling followed by a Scholl oxidation to form a mixture of NGs **73–76**. The authors noted that longer NGs **75** and **76** suffered from incomplete oxidative aryl-aryl coupling. X-ray crystallographic analysis of NG **74** showed that the carbon framework was nonplanar due to steric hindrance in the cove regions, adopting an “up-down” pattern that resulted in **74** being chiral in the solid-state. Not surprisingly, the optical bandgap in going from **73** to **74** shows a decrease as a function of length, with values 2.36 and 1.90 eV, respectively.

**Scheme 17:**
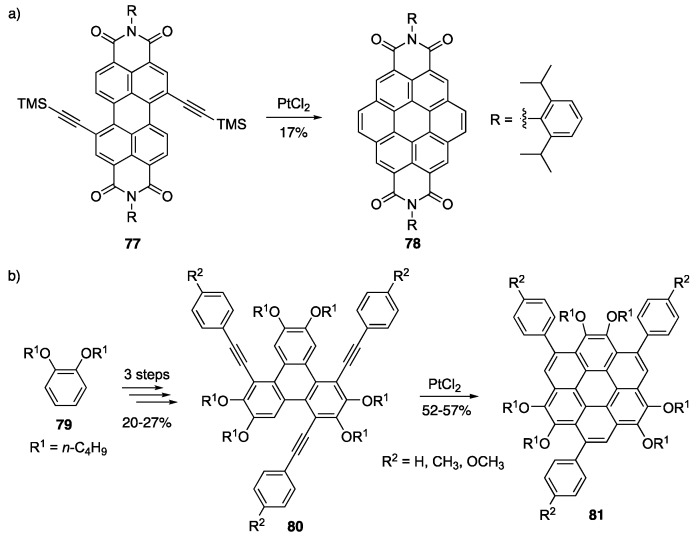
1Pt(II)-catalyzed benzannulation towards functionalized coronenes **79** [96].

Due to their disk like shape and perfect π-conjugation on the outer most rings, coronene derivatives have become of great interest for applications such as discotic liquid crystals and organic electronics [89,90,91,92]. Some of the earliest synthetic routes to make coronenes have been riddled with long procedures and low yields [93,94]. Recently, an effort to optimize this synthesis has been made including the use of transition-metal assisted benzannulation [77,78]. Müllen and coworkers have recently used Pt(II)-catalyzed alkyne benzannulation of **77** with concomitant desilylation to produce coronene tetracarboxdiimide **78** (Scheme 17a) [95]. The synthetic approach towards functionalized coronenes was recently improved by Liu, Yin and coworkers by simplifying the starting material to a functionalized triphenylene moiety **79**, which was converted to their key intermediate **80** in 3 steps (Scheme 17b) [96]. The key step was an alkyne benzannulation step using Pt(II)-catalysis to product functionalized coronenes **81**. These functionalized coronene derivatives have good solubility in common organic solvents due to the presence of alkyl groups around the periphery of the molecule. They have optical energy gaps of 3.02–3.07 eV, showing no photodegradation when irradiated with white light (100 W) for 8 h and only minimal degradation after irradiation with UV light (6 W) after 8 h. 

**Scheme 18:**
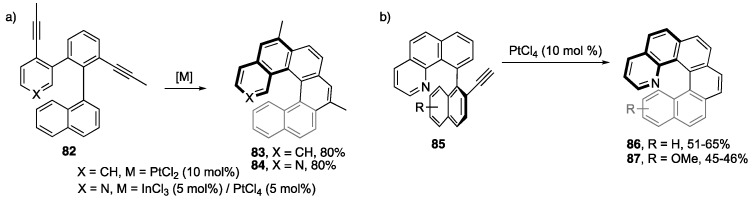
(**a**) [6]Helicene **83** and aza[6]helicene **84** synthesis by Storch and coworkers [99]. (**b**) Aza[6]helicenes **86** and **87** synthesized by Fuchter and coworkers [101].

Various helicenes [97,98], and derivatives thereof, have been produced by Pt(II)-catalyzed alkyne benzannulations [99,100,101,102,103]. For example, the Storch group was able to synthesize [6]helicene **83** in only 6 steps using PtCl_2_ (Scheme 18a) [99]. Interestingly, the synthesis of aza[6]helicene **84** required a mixture of InCl_3_ and PtCl_4_ to effectively promote the alkyne benzannulation [100]. Wanting to extend the substrate scope of aza[6]helicenes, Fuchter and coworkers sought to make functionalized aza[6]helicene derivatives using the same benzannulation conditions as outlined by Storch [101]. They found that the reaction conditions, as reported, yielded very little product, even when up to half an equivalent of catalyst was used. Optimizing of the reaction conditions by changing solvents, increasing the temperature, and switching to PtCl_4_ gave better yields for the conversion of **85** to products **86** and **87** (Scheme 18b). The authors noted that the pre-cyclized atropisomers of **85** (R = H) could be separated by semipreparative chiral HPLC and cyclized to give enantioenriched (*M*)-**86** (90% ee) and *P*-**86** (92% ee).

Surprisingly, the synthesis of large NGs using Au-catalysis is rare but has been successfully employed for alkyne benzannulations to afford various small NGs, including chiral helicene-like compounds [104], pyrenes [71,72,84], and phenanthrenes [69,75,76]. 

**Scheme 19:**
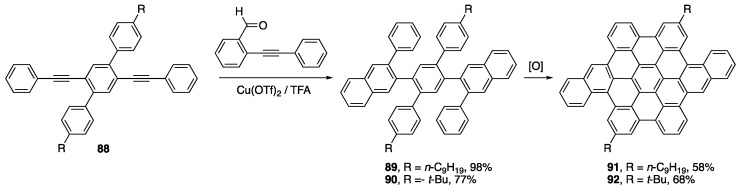
Asao-Yamamoto alkyne benzannulation reported by Dichtel and coworkers towards NGs **91** and **92** [111].

The Dichtel group has recently popularized the Asao-Yamamoto benzannulation [105]—a type of alkyne benzannulation catalyzed by Cu(II) and Brønsted acid that proceeds through a benzopyrylium intermediate—to afford a number of NG precursors that could be oxidized via the Scholl reaction to afford various NGs [106,107,108,109,110,111,112,113]. For example, treatment of diyne **88** under the reaction conditions resulted in a two-fold alkyne benzannulation to afford NG precursors **89** and **90** (Scheme 19) [111]. Compounds **89** and **90** were oxidized under Scholl conditions to afford NG derivatives **91** and **92**. The Dichtel group has also utilized this strategy in a polymeric system to afford GNRs after Scholl oxidation [114]. 

#### 5.2.2. Main Group π-Lewis Acid-Catalyzed Cyclizations

**Scheme 20:**
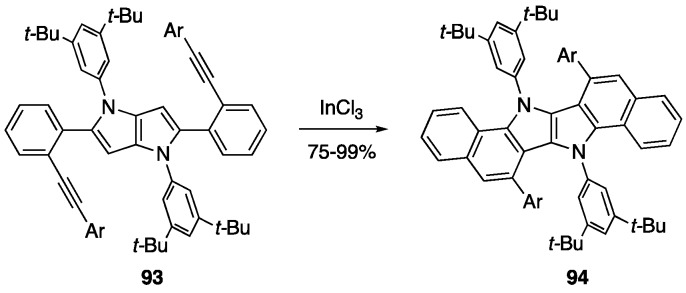
Synthesis of compounds **94** by a two-fold alkyne benzannulation using InCl_3_ [70].

Other metal catalysts have successfully cyclized alkynes to afford a benzene ring, including GaCl_3_ [75,76], SbCl_5_ [115], and InCl_3_ [75,76]. In many cases, InCl_3_ has proven to be superior catalyst in terms of yield for 6-*endo*-*dig* cyclization of alkynes onto heteroaromatics and biphenyls containing haloalkyne substituents [76]. Gryko and coworkers took advantage of this by utilizing InCl_3_ to cyclize both electron-rich and electron-poor ethynylaryl groups onto pyrrolo[3,2-b]pyrroles **93** to afford π-expanded indolo[3,2-b]indole products **94** (Scheme 20) [70]. 

**Scheme 21:**
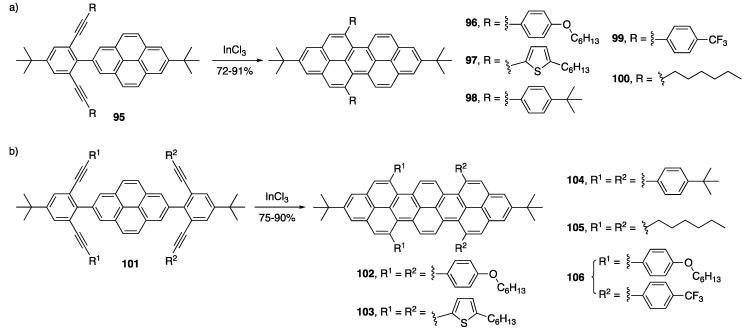
InCl_3_-catalyzed alkyne benzannulation towards a broad scope of (**a**) peropyrenes **96**–**100** and (**b**) teropyrenes **102**–**106** [116].

The Chalifoux group had shown that only electron-rich ethynylaryl substituents successfully cyclize using strong Brønsted acids to give NGs (vide supra). Turning to the work of Fürstner [75,76] and Gryko [70] as inspiration, the Chalifoux group explored the use of InCl_3_ in their two- and four-fold alkyne benzannulation towards the synthesis of peropyrenes and teropyrenes [116]. This catalyst system not only proved to be superior in terms of yield for the cyclization of electron-rich ethynylaryl substituents to produce peropyrenes **96** and **97** but allowed for the cyclization of electron-neutral **98**, electron-poor **99**, and even alkyl-substituted ethynylaryl groups **100** (Scheme 21a). This catalyst system was also able to promote a four-fold alkyne benzannulation to afford a broader scope of teropyrene products **102**–**105**, including the differentially substituted “push-pull” derivative **106** (Scheme 21b). This broader scope of NGs allowed them to study the substituent effects on crystal packing, optical, and electrochemical properties.

**Figure 2:**
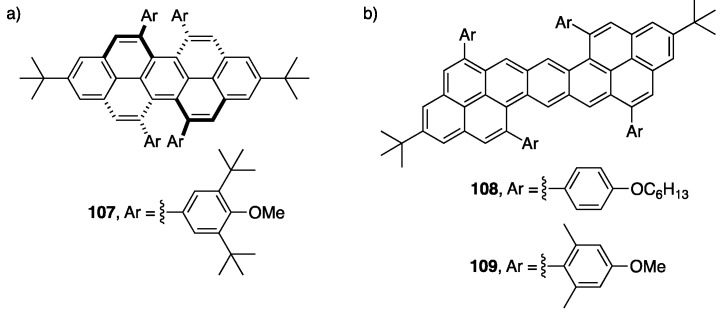
(**a**) Sterically hindered chiral peropyrene **107** [116]. (**b**) BDPs **108** and **109** synthesized by a four-fold alkyne benzannulation [117].

The use of indium(III) catalysts have not only opened the door to a broader scope of NG products available, but also for the cyclization of challenging substrates. Chalifoux and coworkers were able to use InCl_3_ to invoke a four-fold cyclization to afford highly contorted chiral peropyrenes, including the extremely sterically hindered derivative **107** (Figure 2a) [116]. This method proved to be more mild and efficient than what was reported using strong Brønsted acids such as TfOH [48]. The same group recently showed that other NG scaffolds are also accessible using indium(III) catalysis. A four-fold alkyne benzannulation was recently used in the synthesis of benzodipyrene (BDP) derivatives **108** and **109** (Figure 2b) [117]. This reaction required a stronger catalyst system that consisted of InCl_3_/AgNTf_2_ (1:1). The BDP molecules show close stacking of ca. 4 Å in the solid-state, as determined by X-ray crystallographic analysis. The BDPs also show excellent photostability and behave as ^1^O_2_ sensitizers with up to 70% quantum efficiency of ^1^O_2_ generation. 

**Scheme 22:**
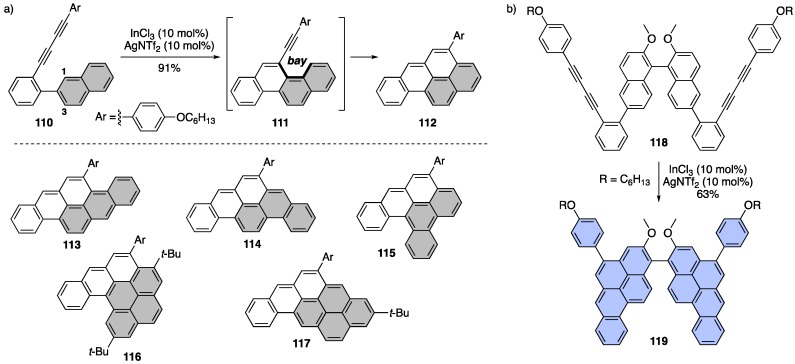
(**a**) Regioselective domino alkyne benzannulation of diynes to form various NGs **112**–**117**. (**b**) Four-fold alkyne benzannulation towards a chiral butterfly ligand motif **119** [118].

Recently, the very first domino alkyne benzannulation of diynes to make a host of irregular NGs was reported by the Chalifoux group [118]. This reaction was shown to be highly regioselective where diyne **110** cyclizes on carbon 1 of the naphthyl group rather than carbon 3, leading to intermediate **111** and a newly formed *bay* region (Scheme 22a). The new *bay* region in **111** is poised for a subsequent alkyne benzannulation to produce NG **112** in excellent yield. Regioselective cyclization onto various other PAH fragments (anthryl, phenanthryl, and pyrenyl) allowed the authors to demonstrate that this is a nice method for arriving at various NGs **113**–**117**. The authors were also able to effect a four-fold cyclization of **118** to make four new carbon-carbon bonds and four new aryl rings in a single reaction to arrive at a large NG-based chiral “butterfly” molecule **119** that could serve as a new chiral ligand scaffold (Scheme 22b).

## 6. Base-Mediated Alkyne Benzannulations

**Scheme 23:**
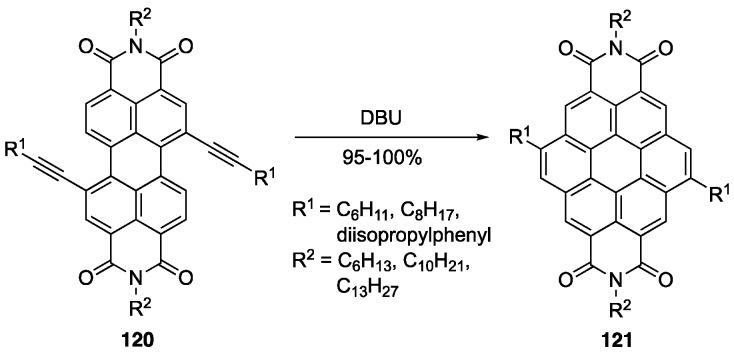
Base-mediated alkyne benzannulations towards CDIs **121** [123].

Coronene tetracarboxdiimides (CDIs) have attracted interest as solar collectors [119] and in lasers [120] due to their chemical, thermal, and photochemical stability. More recently, these derivatives have been of interest for their application in biological systems [121] as well as OLED and OFET materials [122]. Syntheses of these derivatives are lengthy and with poor yields. To optimize the synthetic routes to these CDIs, scientists have begun to use benzannulation methods to grow the number aromatic rings around the perylenediimide (PDI) core. Müllen and coworkers reported the synthesis of CDIs using a DBU-promoted alkyne benzannulation reaction of diethynyl-substituted PDIs **120** to arrive at alkylated CDI derivatives **121** in good yields (Scheme 23) [123]. Since this report, there have been many examples of base-induced alkyne benzannulations to produce various NGs [122,124,125], heteroatom-containing NGs [126], and polymers [127,128,129].

## 7. Conclusions

With the increasing interest in the use of alkyne benzannulation reactions to rapidly extend the π-conjugation towards NG structures, it will be interesting to see what new structures will be presented going into the future. The current toolbox has been expanded to include a number of electrophilic reagents, radical processes and catalysts, many of which offer advantages depending on what core structure is desired. This allows access to a broader scope of NGs, which is highly desirable as their photophysical, chemical, physical and electronic properties can be tuned through variation of their size and structure. With many of the methods discussed here being amenable to the incorporation of various functional groups into the NG and GNR precursors, chemistry now have a way to also “fine tune” the physical properties of these highly desirable materials.
